# Hinders for continued work among persons with fibromyalgia

**DOI:** 10.1186/1471-2474-13-96

**Published:** 2012-06-11

**Authors:** Kaisa Mannerkorpi, Gunvor Gard

**Affiliations:** 1 Department of Rheumatology and Inflammation Research, Institute of Medicine, Sahlgrenska Academy, Gothenburg University, Gothenburg, Sweden; 2 Physiotherapy and Occupational Therapy, Sahlgrenska University Hospital, Gothenburg, Sweden; 3 Department of Health Sciences, Division of Physiotherapy, Faculty of Medicine, Lunds University, Lunds, Sweden; 4 University of Gothenburg Centre for Person-centred Care (GPCC), Sahlgrenska Academy, Gothenburg, Sweden

**Keywords:** Pain, Disability, Fibromyalgia, Work, Qualitative research

## Abstract

**Background:**

Work disability is common among women with fibromyalgia (FM). The aim of the study was to investigate what health problems and work-related difficulties lead to hinders for continued work among women with FM.

**Methods:**

A qualitative interview study. Twenty-seven gainfully employed women with FM participated in five focus group interviews. Their median age was 52 years, ranging from 33 to 62. The transcribed interviews were analyzed by content analysis.

**Results:**

Health problems and work-related demands were identified. Limited physical capacity, increased stress and an increased need of rest were the major health problems, while physical, psychosocial and work organizational demands were the main work-related problems. Personal factors and factors related to family influenced the strategies used to manage the imbalance between the health problems and work-related demands.

**Conclusions:**

Limited physical capacity and an increased need of rest made it difficult for these women to manage the physical, psychosocial and organizational work demands. Adjustment of the work tasks and work environment were the main factors influencing whether the women with FM could work or not.

## Background

Musculoskeletal pain disorders are the main causes of occupational disability and sick leave in Sweden [1,2] and stand for a substantial proportion of the total consumption of health care. Musculoskeletal disorders are common causes of sick leave and disability pensions [3] with a higher prevalence among women than men [3]. The rates of sick leave and stress-related disorders have shown the great increase among women working in health care, teaching, social work, schools, child care and care of the elderly [3].

Fibromyalgia (FM) is characterized by long-lasting, widespread pain and generalized allodynia [4], often accompanied by fatigue, stiffness, nonrestorative sleep and distress. Impaired body function of upper and lower extremities is common [5], and the impairments are associated with activity limitations in daily life [6]. The prevalence of FM ranges from 1% to 3% in the general population [7], and the majority of patients are female. Working women with FM present better health than non-working women [8]. Between 34% and 77% of women with FM are reported to work [9].

Persons with FM usually describe difficulty in managing their work, referring to their symptoms and disabilities. Work ability is primarily a question of a balance between work demands and personal resources [10]. The type of work and the opportunity to adjust the work situation and workload to the reduced physical capacity have been suggested to determine whether or not a person with FM continues to work [9,11]. Deeper knowledge about possible hinders for continued work among women with FM is needed in order to develop adequate strategies to promote work ability. Investigation of hinders that working persons with FM meet at their work is also assumed to contribute with new perspectives to the academic field with interest in these questions. The aim of the present study was to explore hinders for continued work among women with FM.

## Method

Qualitative research approach was chosen to capture and describe the diversity and complexity of the phenomenon selected to be studied. Data was collected by means of focus-group interviews, implying that discussions in a group were used to generate data [12]. Participants invited to focus groups were regarded as experts in the topic chosen to be discussed.

### Selection

The main criteria for inclusion were: gainfully employed women with FM according to ACR 1990 criteria [4], age ranging from 30 to 63 years and ability to participate in the planned interview occasions. The study population was expected to be heterogeneous in terms of working hours and occupations and work places. To further increase credibility, the participants were recruited from three different geographic localizations. The exclusion criteria were other severe somatic or psychiatric disorders and not working for the last three months for any reason.

### Recruitment

Persons fulfilling the main inclusion criteria and living in West Sweden were identified from previous studies in primary health care, and invited to participate in the study. They were contacted by mail with a description of the aim of the focus group study. This was followed up by telephone contact to plan the times for the interviews. The interviews were conducted in three cities. Each person participated once. When the participants arrived, they were asked to fill in a short questionnaire about their demographics, employment and pain impact (Table 1) to verify the inclusion criteria and the heterogeneity of the study population.

**Table 1:** Demographic data, n = 27

		n	%
Education	9 years	6	22
	10-12 years	11	41
	>12 years	10	47
Work status	80-100%	8	30
	50-79%	17	63
	<50%	2	7
Disability pension	75%	1	4
	50%	6	22
	25%	2	7
Employment	Administration	11	41
	Caring occupation	10	37
	Laboratory, industry	3	11
	Sale, gardening	3	11
Living with an adult	Yes	19	70
Born in Sweden	Yes	23	85

### Study group

The study group comprised 27 women with FM. Their median age was 52 years, ranging from 33 to 62. Their median pain impact on daily life, assessed on a 0–10 numeric scale, was 6. Their pain impact ranged from 0 to 10. A total of 41% of the participants worked with administration, 37% with care of children or elderly people, 11% at laboratories or in industry and 11% at sales or gardening. The majority of them worked part-time (70%), while the minority (30%) worked full-time, see Table 1. A large variation of ages, pain impact, occupations and working hours was assumed to assure that various experiences and perspectives were covered, thus strengthening the credibility of the study population.

### Interviews

Interview data were gathered in five focus group interviews, conducted during a period of four weeks, in three cities in West Sweden. The number of participants in each focus group ranged from four to eight. To achieve dependability of the interviews, the interviews were initiated by the moderator (the first author), who described the goal of the study which was to search for deeper knowledge about health problems and difficulties leading to hinders to or difficulties in managing employment. Second, the moderator guided the discussions following an interview guide with open-ended questions covering the following themes: how the respondents experienced their work, what factors influenced their work load and what factors could lead to hinders or difficulties. Third, interactions between the participants were encouraged. Fourth, at the end the moderator summed up the discussions inviting the participants to add new things or confirm or clarify any aspects discussed. Fifth, a co-moderator recorded the interviews and monitored the interview process. The interviews lasted for two hours. When all the interviews were conducted, they were transcribed verbatim by the co-moderator.

### Analysis

The interviews were read separately by the two researches to get a sense of the whole material. One of the researches had acted as the moderator during the interviews, while the other based her analysis on the transcripts. Whole interviews built the unit of analysis; no parts were excluded from the analyses. The content analysis [13] started by identification of meaning units, jointly traced and analyzed by the two researchers in each individual protocol. The research question exploring hinders for work steered the way the meaning units were discerned. The meaning units were condensed, abstracted and labeled with codes [14], separately by the two researchers. The codes were compared and sorted into sub-categories and categories. The analysis moved back and forth through these steps [14] and tentative sub-categories and categories were discussed and elaborated and negotiated until agreement was reached (see Table 2). Citations to illuminate the categories were selected. Co-operation between the two researchers was assumed to increase the credibility of the analysis.

**Table 2:** The three categories identified with the subcategories

Categories	Subcategories
Work-related demands	Physical work demands
	Psychosocial work demands
	Work organizational demands
Health problems	Limited physical capacity
	Increased stress
	Increased need of rest
Context-related factors	Personal factors
	Family related factors

### Ethics

The study was approved by the ethics committee at Göteborg University. Written and verbal information was given to all the participants, and written consent was obtained from all patients.

## Results

The interviews were first analysed separately and then jointly by the two authors, and the independently developed codes and categories were found to be very similar.

Problems related to health and work were identified. Limited physical capacity, increased stress and increased need of rest were found to constitute the main health problems (Table 2). There was an imbalance between the participants’ reduced resources due to their health problems and the work-related demands, which were physical work demands, psychosocial work demands and work-organizational demands. Personal and family-related factors influenced the strategies for managing the imbalance between the health problems and the work-related demands. See Table 2.

### Work-related demands

#### Physical work demands

Several participants described that their physical workload resulted in increased pain, fatigue and exhaustion. A worsening of the symptoms was related to long work days, lengthy static work, lengthy walking, static work with the arms, and lifting or carrying heavy objects. High-precision work with manual instruments was also perceived as physically demanding, resulting in increased pain, as well as physiological factors, such as a low outdoor temperature or a high sound level from machinery.

"“When I carry things I get terrible pain in my arms.”"

Other participants said that their chronic pain did not change with work.

"“I have this constant pain in my shoulders…//…it doesn’t get better or worse, so to speak.”"

Physical work demands could be prevented to some degree by careful planning, for example by minimizing the number of lengthy walks by careful planning of daily work tasks, by using an elevator or by asking colleagues for help with heavy work tasks.

"“I say (to a workmate): Can you do it today, because I can’t manage it.”"

A high physical work demand was an inability to take pauses to alleviate health problems. However, this appeared to be an accepted condition in work in health care:

"“I can’t take pauses, no, I can’t leave the children’s group.”"

#### Psychosocial work demands

A high psychosocial workload in terms of time pressure, low ability to control work and unclear work tasks increased insecurity and stress. Difficulties in fulfilling performance demands were also stressful.

"“Pressure and stress, you can´t influence anything by yourself, you have to accept it.”"

Most of the participants experienced little social support from their supervisors, which increased their feeling of stress during periods when they did not manage their work according to expectations.

"“My supervisor said that it isn’t our responsibility to look after your illness, it’s the Swedish Social Insurance Agency’s, this is stressful for me, I feel that when I’m feeling ill and can´t manage to go to work, I have a bad conscience because I know what his opinion is.”"

Other participants described having an understanding supervisor with whom they could plan their work schedule during the periods they were more ill. The experience of support appeared to strengthen their sense of confidence in being able to manage their employment despite their disabilities.

"“I have a good situation, I can call my supervisor and say I have to come to work a little late today, I can´t manage to get out of bed just now. (The supervisor said:) No, come when you want!”"

#### Work organizational demands

Most participants had gone from full-time work to part-time, 75% or 50%, by their own choice or in negotiations with their employers, which negatively affected their private economy. Some participants had changed stressful full-time work for less stressful work.

"“I work 75%, I was forced to reduce my working time by 25%, I couldn’t be sick-listed any longer and I couldn’t manage to work more (full-time).”"

A schedule that is common for healthy individuals, that is, work for five consecutive days a week, was perceived as stressful. Some participants had reduced their daily working hours from eight to four but still experienced a high workload and time pressure and did not manage to accomplish what was expected during these four hours, which increased their feeling of stress.

"“I work 8–12 three days a week and one day eight hours, it’s very stressful to work 8–12, you have to stop working when you’ve just begun, and I tense up, I never feel that I finish work, and I don´t get a rest either, you never have a day of relaxation from work.”"

As the severity of the symptoms fluctuated, an inflexible work schedule, with long working days and no opportunity to influence the schedule, increased their stress. Some described a need of a day off in the middle of the week to get some rest. Participants who had reduced their work week so that they worked eight hours a day, but had one day off in middle of the week, appeared to manage their work week better. They used the “free” day to rest and gather energy for the rest of week.

"“Now I work every other day, Monday, Wednesday and Friday one week and Tuesday and Thursday every second week. If I had to work between 8 and 12 every day I think I’d have a headache and pain all the time.”"

Some participants described having flexible working conditions, being able to start later in the morning on days when they had a great deal of pain, which appeared to have diminished their feeling of stress and pressure.

"“Getting up early in the morning is difficult…I’ve had very a flexible system for a period…if I couldn’t start at 10 I tried at 12. I came to work, but not at the same time every day.”"

Downsizing and organizational changes also increased stress.

"“It is very stressful to have a manager that doesn´t understand anything and doesn´t care. You have to simply sit there and perform 100 percent.”"

### Health problems

#### Limited physical capacity

Most of the participants described limited physical capacity relative to the demands of their work. They lived with a conflict between their willingness to work and their reduced physical capacity. Their reduced physical capacity could lead to difficulties accomplishing full-time work or accomplishing their work tasks in time. Those who had to work long workdays experienced fatigue and exhaustion after work.

"“I am totally exhausted after nine-hour working days. I lie in bed a whole day afterwards …if I work five hours a day and five hours the next day, then I can keep going, there’s a difference.”"

The limited physical capacity could lead to emotional exhaustion and overload. Some participants experienced that they had to use their maximal physical capacity to accomplish their usual work tasks, which made them feel more or less emotionally exhausted. The emotional overload was associated with a high level of pain. They described that when their pain reached a certain level, they were not able handle it, but felt exhausted, irritated, insufficient, depressed or angry.

"“When the pain goes over a certain limit, I can become a little… aggressive..//..I can answer in a way that I wouldn’t otherwise…//…like today, I got angry… and that’s not good…//… so it affects me emotionally.”"

#### Increased stress

The limited physical capacity leading to exhaustion and an increased perception of stress could temporarily lead to cognitive problems and overload. Some respondents described how high psychosocial stress at work resulted in an overload that impaired their thinking and memory. Cognitive overload was experienced during the workday or after coming home. Such overload was experienced as incurring fear. Cognitive problems such as impaired concentration and increased fatigue were also related to dysfunctional sleep.

"“I’m totally exhausted some days…and it’s like my brain stops working, and that’s the thing I think is worst, that logical thinking disappears.”"

#### Increased need of rest

Fatigue and an increased need of rest negatively influenced their work ability. The participants described a need of rest during the daytime and after completing the day´s work. Because of fatigue, they lacked the energy to socialize and participate in activities at home and/or during leisure time. It appeared that a large part of leisure time was used to rest in order to manage being able to work.

"“I have to lie down when I get home from work. Yes, I can fall asleep immediately, I’m chronically fatigued.”"

Some participants needed a period of sick leave to get enough rest. Most of them worked part-time and had been sick-listed during periods of extreme exhaustion and pain, as they were not able to manage work during those periods. The participants’ need of rest had been met by the Swedish Social Insurance Agency in different ways: by a period of sick leave or time-limited disability pension.

"“I have time-limited early retirement pension. I’ve done everything they (the Swedish Social Insurance Agency) asked me to do, I’ve tried and tried and tried, but there’s just a stop (in work ability).”"

### Context-related factors

#### Personal factors

Personal factors influenced the strategies that were chosen to manage the imbalance created by the health problems and work demands. Several participants described a high ambition and motivation to work in spite of the difficulties they experienced.

Some participants described difficulties in setting limits, wanting to manage all their work tasks, although the workload that was expected of them appeared to be beyond their abilities. Others described being afraid of conflicts at work. Overload resulted in more pain.

"“I don´t want to say to my manager that I can´t manage this, that I need some help, so I try and I clench my teeth and I ache all over…..but I do the job and when I come home I collapse and take strong medication for the night to manage the next day.”"

A strong motivation to accomplish as much as healthy individuals do appeared to be a social norm that several participants tried to fulfill, but failed, which may be a reason for their high level of stress.

"“I’ve always felt that when I’m at work I have to perform as much as a healthy person does, and …so I kept at it and had pain and still kept at it and got sick. I had to start with working four hours a day. But still, when I was at work, I wanted to perform well because I didn´t want to be worse than anyone else…anyway, I think that’s where my stress comes from.”"

#### Family-related factors

Family-related factors influenced the strategies the women had for managing the imbalance between their health problems and work demands. The participants described both positive and negative influences of family life on their work ability, well-being and functioning. Some participants experienced great responsibility for relatives who were sick. They described a decreased energy because of the responsibility they felt for sick parents or because of their parents´ lack of understanding of their limitations. The participants who had young children had less time to rest and relax after work.

"“I can’t get home and rest because my children are at home in the afternoon, and they do their homework, and there are friends and afternoon snacks. I can’t ever go and lie down during the daytime, so I’m very tired when I get into bed in the evening.”"

In general the participants experienced a restriction in their social life and that their family had taken on greater responsibility for household tasks.

"“The family gets hurt or has to struggle because you don´t manage to do much at home.”"

Many participants had altered their activities and priorities in life to manage their work. They described having excluded several activities at home as well as social activities because of fatigue or as a preventive strategy, in order not to drain themselves of energy. Even very pleasant social activities could be experienced as increasing stress and were thus excluded.

"“I don´t want to socialize with anyone when I come home after work … I want to be left alone… I’m tired.”"

## Discussion

A limited physical capacity, increased stress and increased need of rest were the main health problems that made it difficult to manage work. The participants described an imbalance between their reduced resources due to their health problems and the work-related demands, which were physical work demands, psychosocial work demands and work-organizational demands. Influence of work on health varied. While several participants perceived great difficulties due to increased pain and other symptoms, others did not feel that their health problems were influenced by work.

Work ability can be seen as a balance between a person´s resources and the demands of the work that she performs [10]. Both physical and mental resources were found to be limited among participants in the present study. Some participants used their maximal capacity to manage their work, describing how their pain could at times increase to such a level that it led to emotional exhaustion, which was expressed in irritation, depression, aggression or a loss of clarity of thought. The participants appeared to be aware of causes and consequences of overload and tried to handle them in the workplace. Our findings confirm those of previous studies that report impaired physical capacity [6], cognitive dysfunction [15] and decreased work ability in FM [9], and provide a detailed description of the patient’s perspective of how physical overload progresses to cognitive overload. It appears to be important to identify situations that lead to cognitive overload, both for the employer and the employee. Greater control of work and better self-efficacy and self-confidence might put a limit to this type of overload and its consequences.

Here, an unclear definition of work tasks was a common element of the psychosocial workload, and it is probably a common characteristic of many occupations in the areas of service and care. The high level of psychosocial stress described by the participants agrees with earlier research that has shown that time pressure, poor control and unclear work roles lead to stress and low work satisfaction [16].

An interesting finding was that a shortening of the workday from eight to four hours did not always result in a lesser workload, which is commonly expected by the physician and the employee. The interviews showed that if high psychosocial work demands remained, such as unclear content and the number of work tasks, psychosocial stress did not diminish when the work day was cut to four hours. Furthermore, once the stress level had increased, it appeared to maintain for the rest of the day. This indicates the importance of defining clear work roles and individual responsibilities.

In this context, the importance of the supervisor’s attitude is understandable. Our sample claimed that the supervisor had the main influence in terms of how work was scheduled and how work tasks were defined, which either broadened or limited the ability of the participant to control her work situation. This confirms earlier studies of the importance of feedback and social support given by supervisors and colleagues at work for continuing to be healthy and able to work [17,18].

A previous five-year longitudinal study of working women with short-term FM showed that work did not prevent a decline in health status over time [8]. On the basis of our results, it is likely that person who experiences heavy physical or psychosocial workload risks deterioration in her health and work ability. The participants in our study who had succeeded in adjusting their work tasks and the pace of work to their daily symptoms and abilities appeared to have the best possibilities for maintaining their health and employment. These findings confirm previous reports that indicate that the individual´s work ability is dependent on such factors as coping strategies, work climate, work demands, attitudes and values [19].

Rest and relaxation appeared to be the primary strategies used to recover for the next workday, and the ability to find the time to rest was in part dependent on factors related to the family, such as having responsibility for small children or sick parents and whether they received support from their significant others.

From a health promotion perspective, it appears that employers, employees with FM and health care professionals should cooperate to develop the work environment by adjustments of the work demands. The interviews indicated that many persons with FM were able to take an active role to improve their work ability. Workers with FM who had an opportunity to vary their work positions and work tasks during the day and to take short breaks managed their work better. These changes would also enhance “pacing”, which is an often recommended strategy for patients with FM to be able to manage daily tasks [20] but which was seldom possible to apply in work situations, as described by our sample. To adjust the work situation to match the ability of persons with FM, the heavy physical tasks, frequent carrying and lifting, static movements, dynamic repetitive work and eccentric muscle work need to be limited, as has also been reported previously [9]. Adjusting work hours from day to day and modifying work tasks according to the person’s actual resources appear to be helpful strategies for patients with FM so that they can continue to work in the long term. Besides these factors, the life situation as a whole and the physical and psychosocial work environment may determine whether a person can remain at work.

Selection of the study population aimed to reach homogeneity based on shared experience of FM and being active in work life, while variations in age, pain impact, occupations and work hours brought variations of experiences and perspectives. Heterogeneity of the study population was found to enrich the discussions. Selection of the study population is assumed to strengthen the credibility of the data analyzed. Focus-group interviews was a suitable method for data sampling, as the participants openly shared their experiences and reflections with each other during relaxed discussions, also found in previous studies using the same method [21]. Measures to achieve consistency of data collection included a short data gathering period (four weeks), standardized start and completion of the interview session, use of the interview guide, and the role of the co-moderator. The standardization allowed possibility to deepen the interviews of the topics discussed. These methodological aspects are supposed to strengthen dependability, an aspect of trustworthiness in data collection.

The first author acted as moderator in all the focus groups, while the second author based her analysis solely on the transcribed interviews. This methodology brought different perspectives on the analysis. The interviews were analysed separately and jointly by the two authors, moving back and forth through coding and categorization [14]. The independently developed codes and categories were very similar, which is expected to increase the credibility of the interpretations, an aspect of trustworthiness of the analysis.

A limitation of the qualitative research approach is the fact that the results cannot be generalized. It is possible that gainfully employed women with FM in other settings and with other living conditions have experienced other barriers for professional work. There is always a possibility that new categories might be identified in an interview with a new study population. As authors we consider that the results are relevant when planning rehabilitation for women with FM or other long-lasting pain disorders, but transferability of the results to other contexts has to be judged by the reader [14].

## Conclusion

Limited physical capacity, increased stress and a greater need of rest were found to be the main health problems that influenced work ability in patients with FM. Because of their health problems, most of the participants found it difficult to manage physical, psychosocial and organizational work demands. Opportunities to vary and adjust the work tasks and work environment were the main factors influencing whether the women with FM could work or not.

## Misc

This work was supported by The Swedish Research Council, the ALF at Sahlgrenska University Hospital, the Research and Development Council of Göteborg and Södra Bohuslän, Västra Götaland Region, Sweden

## Competing interests

No financial and non-financial interest exists, which could create a potential conflict of interest with regard to the work.

## Authors’ contributions

KM conceived the study, recruited the participants and conducted the focus-group interviews. Both authors (KM,GG) together worked out the study design, analyzed the data and drafted the manuscript. Both authors approved the final manuscript.

## Pre-publication history

The pre-publication history for this paper can be accessed here:

http://www.biomedcentral.com/1471-2474/13/96/prepub
